# Effective Synthesis of mRNA during In Vitro Transcription with Fewer Impurities Produced

**DOI:** 10.3390/molecules29194713

**Published:** 2024-10-05

**Authors:** Wei He, Qi Geng, Guiying Ji, Ji Li, Dan Wang, Yucai He, Qiuheng Jin, Jianren Ye

**Affiliations:** 1 College of Forestry, Nanjing Forestry University, Nanjing 210037, China; wwh09006@163.com; 2 Vazyme Biotech Co., Ltd., Nanjing 210037, China; gengqi@vazyme.com (Q.G.); jiguiying@vazyme.com (G.J.); lijikelly@163.com (J.L.); wangdan0301@vazyme.com (D.W.); 3 School of Pharmacy, Changzhou University, Changzhou 213164, China

**Keywords:** mRNA, in vitro transcription, impurities, dsRNA, mRNA integrity

## Abstract

The remarkable efficacy of COVID-19 vaccines has established mRNA as a highly promising biomedical technology. However, the adequate application of mRNA therapeutics necessitates additional measures to mitigate the inherent immunogenicity, which is predominantly caused by dsRNA. As a byproduct of the in vitro transcription of mRNA, dsRNA was reported to be originated through several distinct mechanisms, including the extension of 3′ loop-back hairpins, the extension of hybridized abortive transcripts, and promoter-independent transcription. The intricate mechanisms involved pose a dilemma as the reduction in dsRNA results in a concomitant decrease in other critical quality attributes of mRNA. Here, we demonstrate that the promoter binding motifs of T7 RNA polymerase directly impact the production of promoter-independent transcription-based dsRNA. Specifically, the G753A mutation significantly reduces the formation of dsRNA byproducts, which can further combine with modified nucleotides to enhance the effectiveness of dsRNA mitigation and with previously reported high-integrity mutation K389A to minimize side effects. Accordingly, the present study reports a cost-effective approach to synthesize high-purity, less immunostimulatory mRNA by using an engineered T7 RNA polymerase mutant.

## 1. Introduction

In recent years, messenger ribonucleic acid (mRNA)-based therapies have presented a strong development trend [1,2,3,4]. The tremendous potency of mRNA drugs, notably mRNA vaccines, helps them fight various diseases including but not limited to infectious, rare, and malignant neoplastic diseases; they can be used at extremely low dosages for greater security, allowing for amazingly rapid drug discovery and large-scale development [5,6,7,8,9]. mRNA vaccines have achieved extraordinary success, especially against COVID-19.

**Figure 1:**
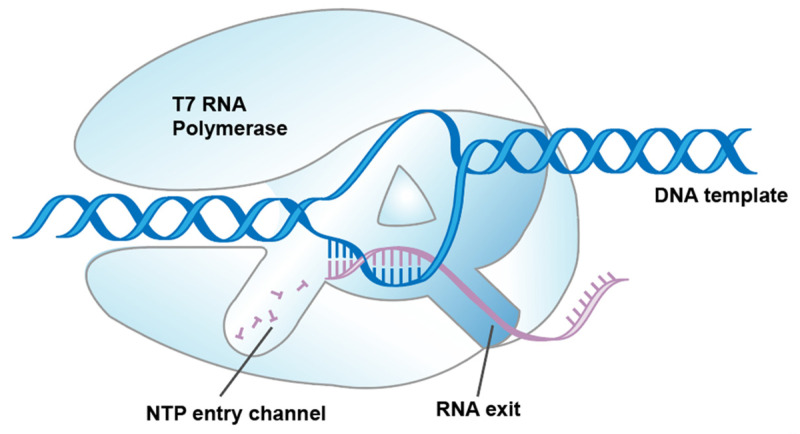
Schematic illustration showing T7 RNAP-transcribed RNA synthesis.

In general, mRNA drugs are synthesized in vitro by RNA polymerases. T7 RNAP, encoded by the T7 virus (bacteriophage), is the best characterized member of the widespread family of RNAPs [10]. Hitherto, mRNA vaccines approved by the FDA have been transcribed by T7 RNAP in vitro [11]. Compared with eukaryotic RNA polymerases and multi-subunit prokaryotic RNA polymerases, T7 RNAP is one of the simplest enzymes that transcribes RNA through a single subunit composed of multiple functional subdomains (Figure 1) [12,13,14].

T7 RNAP undergoes transitions from the initiation phase, where it binds with the T7 promoter to initiate de novo mRNA synthesis, to the elongation phase, where mRNA products are released and byproducts are unavoidably generated during the complex conformational transitions [15,16,17]. There are two impurities that have been mainly focused on in previous research:

Firstly, double-stranded RNA (dsRNA) is a key immunogenic byproduct which acts as a major trigger activating pattern recognition receptors and innate adverse immune responses [18,19,20,21]. Complex downstream column purification is the traditional method used for dsRNA removal [22,23]. It is extremely difficult to decrease the dsRNA content using this method due to the similar structure of mRNA products with the byproducts of dsRNA retained in the products synthesized during in vitro transcription. It was reported that using modified nucleic acid N1-methylpseudouridine (m1ψ) as a substrate was a valid method for dsRNA reduction [24]. mRNA vaccines designed for infectious diseases like BNT162b2, the first vaccine approved by the FDA, use m1ψ as the substrate to decrease immunogenicity [25,26,27]. Because of this innovation, Karikó and Drew Weissman won the Nobel Prize in Physiology or Medicine last year [24,28,29,30]. As we used mRNA vaccines to fight against rare or malignant neoplastic diseases, the rate of dsRNA reduction by m1ψ was probably not enough due to the multiplying increase in dosage [31]. Recent studies have demonstrated that T7 RNAP mutants have the capacity for dsRNA reduction. Binding T7 RNAP mutants with m1ψ might be an effective method for further dsRNA decreases.

Except for dsRNA byproduct production, fragmented mRNA occupied the largest proportion of impurities produced during in vitro transcription [18,32,33]. Fragmented mRNA mainly occurs in two ways: One way is the hydrolysis of mRNA products, which could be accelerated by the cofactor of T7 RNAP- Mg^2+^; in in vitro transcription, the presence of hydrated magnesium ions not only initiates the cleavage of RNA by deprotonating the 2′-OH group but also stabilizes the transition states and intermediates involved, enhancing the efficiency and specificity of the degradation reaction [34,35,36,37,38]. The second is during T7 RNAP-catalyzed transcription, where various factors can lead to the premature termination of mRNA synthesis. This can result in truncated or fragmented mRNA products that may not be functional or may possess altered properties compared to full-length mRNA [33].

**Scheme 1:**
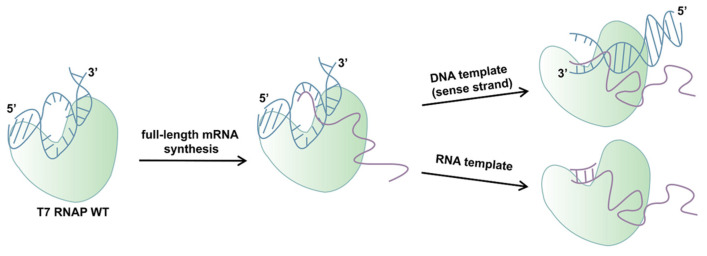
A schematic illustration showing the dsRNA generation catalyzed by T7 RNAP WT.

In this work, we aimed to further explore the mechanisms of impurity generation, especially dsRNA byproducts in mRNA production transcribed in vitro by T7 RNAP (Scheme 1). Based on previous research, the C-helix is related to the yield of RNA products in promoter-independent transcription, which generates full-length dsRNA. The specificity loop plays a critical role in DNA template-specific recognition and in the initiation of transcription, which may have a connection with promoter-dependent transcription and further be related to dsRNA production. Therefore, control of dsRNA from the origin may be achieved through in-depth research on the specificity loop. In addition, we studied the integrity of mRNA products catalyzed by the mutant which not only decreased dsRNA production but also eliminated the negative impact on the integrity of the transcription products.

## 2. Results and Discussion

### 2.1. Screening Specificity Loop Substitutions via Conservative Analysis

Recent studies have demonstrated that the design of T7 RNAP was an effective method for dsRNA reduction. For instance, promoter-independent transcription produces long-stranded dsRNA [39,40]. T7 RNAP C-helix mutants were reported to reduce promoter-independent transcription but DNA terminus-dependent transcription and exhibited a significant decrease in dsRNA byproducts [40]. The C-helix (aa 28–71) was from the N-terminal domain of T7 RNAP, which plays a major role in sequence-specific promoter binding and opening [41]. The C-helix is also important for maintaining transcription complex stability from initiation to elongation. Based on previous research, controlling promoter-independent transcription is necessary for reducing dsRNA generation [42]. Similar to the C-helix influencing promoter-independent transcription, the specificity loop (aa 740–769) located on the C-terminal domain of T7 RNAP proved critical for T7 RNAP-specific T7 promoter recognition and may further impact the rate of promoter-independent transcription [43,44]. Thus, specificity loop mutants of T7 RNAP, which influences specific recognition and affinity with the T7 promoter, may also have the ability to reduce dsRNA in in vitro transcription.

**Figure 2:**
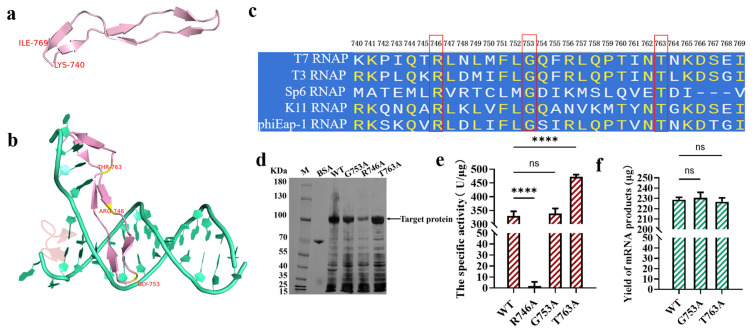
The detection of the site G753 from the specificity loop of T7 RNAP (the error bars represent ±SD; **** *p*  <  0.0001). (**a**) The structure of the specificity loop; (**b**) a structural analysis of the specificity loop binding with the promoter (green: DNA template; pink: the specificity loop) (PDB: 1CEZ, panels (**a**,**b**) were generated by using PyMOL Version 3.0); (**c**) the sequence consistency of T7, T3, Sp6, K11, and phiEap-1 RNAP (the panel was generated by using SnapGene Version 4.2.4). (**d**) an SDS-PAGE analysis of the overexpression of the protein (**e**) The measurement of the specific activity of the enzymes. (**f**) The yield of mRNA products transcribed by the T7 RNAP WT/G753A/T763A.

T7 RNAP is homologous to the DNA-directed RNA polymerases from Enterobacteria phage T3 [45], SP6 [46], K11 [47], and phiEap-1 [48]. DNA-directed transcription is initiated from recognizing and discriminating among similarly homologous but different promoter sequences. The specificity loop of T7 RNAP extends from residues 740 to 769 and is a crucial functional structure that distinguishes the T7 promoter from other homologous promoters [10,44] (Figure 2a,b). Thus, most residues from the specificity loop are not conservative for recognizing specific sequences and then initiating transcription. For instance, when N748 was replaced by amino acid D, the T7 RNAP mutant changed to specifically recognize the T3 promoter; the T7 RNAP variant R756A was inactive due to the hydrogen-bonding interaction being lost between the guanidinium group of R756 and deoxyribonucleic acids [49].

A conservative analysis of the specificity loops of T3, SP6, K11, and phiEap-1-like phage RNAPs revealed that only a few sites were highly conserved, like R746, T763, and G753 (Figure 2c). The side chain of the amino acid Gly was -H, which does not easily form hydrogen bonds or other bonds with the promoter to directly participate in promoter recognition. R746 has a guanidine group, which easily forms hydrogen bonds, similar to T763, which has the -OH group. Thus, R746 and T763 may participate in promoter recognition. We used the traditional method of employing amino acid alanine (A) instead of the original amino acids for our research [50]. Firstly, the protein expression of the T7 RNAP WT and the variant was analyzed using SDS-PAGE. There was no significant difference in protein expression among the T7 RNAP WT, G753A, and T763A (Figure 2d), while R746A substitution decreased protein expression. The enzyme activities were then determined. As shown in Figure 2e, there was no significant difference in activity between the T7 RNAP WT (330.3 ± 16.34 U/μg) and G753A (338.7 ± 18.54 U/μg). The specific activity of T7 RNAP T763A was improved by more than 40% at 37 °C, while T7 RNAP R746A was inactive. We inferred that R746 may play an important role in recognizing the promoter, while the connection between T763 and the promoter is redundant. Thus, T7 RNAP R746A lost the ability to initiate transcription, while T763A subtracted the redundant bond, which markedly improved the catalytic activity (Figure 2e). As shown in Figure 2f and Table S1, by adding the catalysts T7 RNAP G753A/T763A into the reaction of in vitro transcription, 230.69 ± 5.24/226.75 ± 3.87 μg of RNA products was acquired, which is similar to the yield of the T7 RNAP WT (228.80 ± 2.34 μg). Mutants such as G47W and G47A+884G, which were used for dsRNA reduction in previous studies, can more or less decrease the yield of mRNA products [40,51]. Thus, modifications in the specificity loop have more economic benefits for further research on decreasing dsRNA byproducts.

### 2.2. T7 RNAP Substitutions Closely Related with the Production of dsRNA Byproducts

dsRNA byproducts can be triggered by promoter-independent transcription. While the specificity loop plays a major role in promoter-dependent transcription, it has a strong possibility of influencing the production rate of dsRNA byproducts. The yield of dsRNA byproducts in in vitro transcription by the T7 RNAP WT was much less than 1% (μg/μg). Even though dsRNA was present in trace amounts, the immunogenic side effects caused by dsRNA cannot be ignored [20,21].

**Figure 3:**
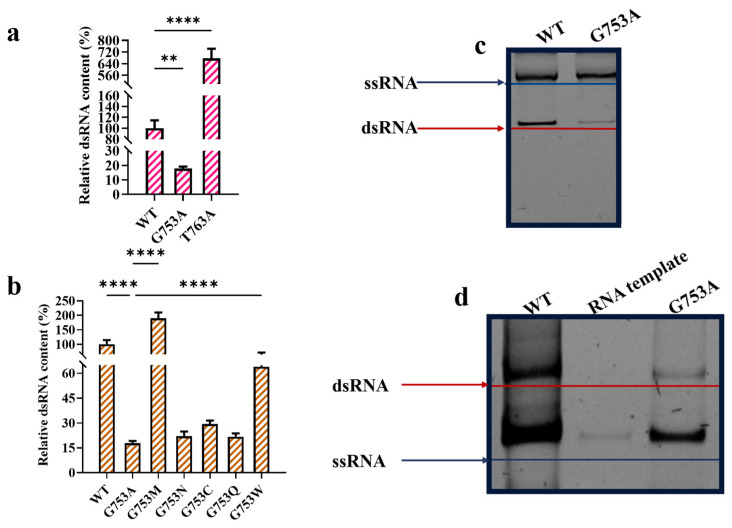
T7 RNAP G753 reduced dsRNA production in in vitro transcription (error bars represent ±SD; ** *p*  <  0.01, **** *p*  <  0.0001). (**a**) Relative dsRNA content (%). (**b**) Site G753 related with dsRNA production. (**c**) T7 RNAP G753A reduced antisense RNA production initiated from promoter-less DNA end. (**d**) T7 RNAP G753A reduced antisense RNA production, which was synthesized using RNA as template (note: short RNA sequence length (≤150 nt) causes ssRNA to migrate more quickly than dsRNA of equivalent size; longer sequence length (512 nt) causes ssRNA to migrate more slowly than dsRNA of equivalent size [39]).

Compared with the T7 RNAP WT, T7 RNAP T763A significantly increased dsRNA impurities by six times (Figure 3a). Meanwhile, T7 RNAP G753A-transcribed mRNA obviously decreased the yield of dsRNA byproducts. The rate of dsRNA production among mRNA products catalyzed by the T7 RNAP WT was plotted at 100%. T7 RNAP G753A-catalyzed mRNA products reduced dsRNA impurities to 17.76 ± 1.37% (Figure 3a). To further confirm whether position 753 from the specificity loop is related to dsRNA production, we conservatively mutated G753 to neutral amino acids N, C, Q, M, and W without forming extra charge bond forces with negatively charged RNA. As shown in Figure 3b, except for G753M, all other mutations reduced dsRNA production when catalyzed by T7 RNAP, with G753A showing the strongest effort and the aromatic amino acid replacement G753W showing the weakest effort. This result demonstrates that our previous intuition was right: the specificity loop strongly relates to dsRNA production. T7 RNAP G753A has the potential to industrially synthesize mRNA with a lower production of dsRNA byproducts.

To better analyze the causes of dsRNA reduction catalyzed by T7 RNAP G753A, further research on its mechanisms was needed. Mu X et al. confirmed that T7 RNAP-transcribed antisense RNA is created by using the sense strand of DNA as the template and begins at the 3′ end [39]. Firstly, we used this method to verify whether T7 RNAP G753A reduced the formation of antisense RNA transcribed using the sense strand of DNA as the template. As shown in Figure 3c, T7 RNAP G753A significantly reduced the sense strand of DNA template-caused antisense RNA production, confirming that substitution from the specificity loop triggered the reduction in antisense transcription from the promoter-less DNA end.

Apart from antisense RNA generation from another strand of the promoter-less DNA template, RNA products also have the possibility of being templates to initiate transcription [52]. We used a short length of mRNA to replace DNA as the template and found that the T7 RNAP WT catalyzed a large amount of antisense RNA. While being replaced by T7 RNAP G753A, most antisense RNA was reduced via the RNA template (Figure 3d). We inferred that T7 RNAP G753A impacted the stability of the complex formed by non-specific binding so that the rate of the 3′ terminal combination without promoter recognition was reduced. Thus, the yield of dsRNA decreased due to the reduction in trigger antisense RNA.

### 2.3. m1ψTP Further Decreases Production of dsRNA Byproducts

**Figure 4:**
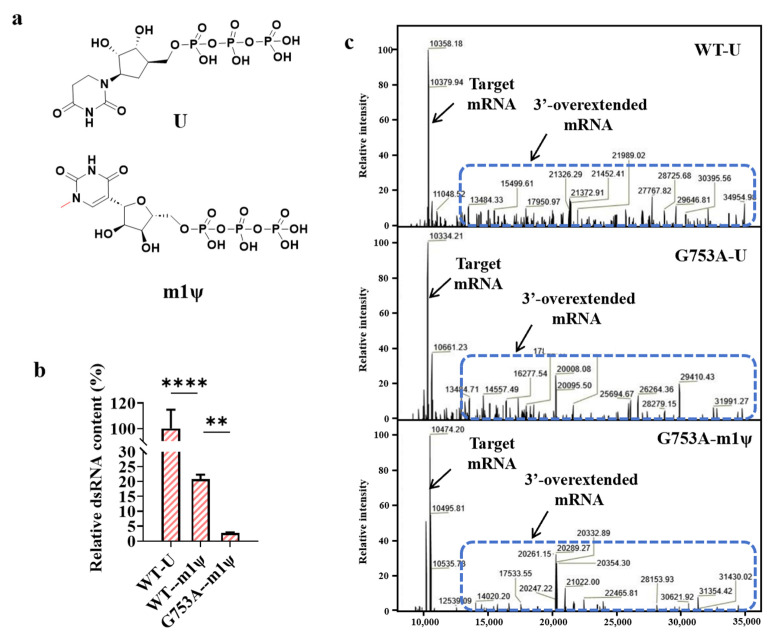
m1ψ further decreased dsRNA production in in vitro transcription (the error bars represent ±SD; ** *p*  <  0.01, **** *p*  <  0.0001). (**a**) The chemical structures of U and m1ψ. (**b**) The relative dsRNA content (%). (**c**) A schematic diagram showing dsRNA derived from overextended mRNA. Mass analytics of mRNA production.

As we described above, using the modified nucleic acid m1ψ (the structure is shown in Figure 4a) as a raw material for mRNA transcription also reduced the production of dsRNA byproducts. We used m1ψ to replace uridine as the substrate and used T7 RNAP G753A as the catalyst for mRNA synthesis in vitro, and the yield of dsRNA byproducts was further decreased to 2.74 ± 0.18% (Figure 4b). Nance K D et al. found that m1ψ can reduce antisense RNA production, while we inferred that m1ψ probably triggered other mechanisms of reducing dsRNA production, which is why the ratio of dsRNA content was further decreased sharply [53]. As previously reported, the 3′ overextension of RNA was another major trigger of dsRNA production, and there are already several methods used to reduce the 3′ overextension of RNA, such as high-temperature transcription and the addition of chaotropic agents [54,55]. The use of mutant T7 RNAP G47A+884G also reduced the generation of 3′-overextended RNA but significantly reduced the yield of mRNA [51].

We analyzed the rate of 3′-overextended RNA generation by using LC-MS; interestingly, as shown in Figure 4c, the mutant T7 RNAP G753A also reduced impurities of the 3′-overextended RNA, but a large amount of 3′-overextended impurities were still produced. When binding with m1ψ, most 3′-overextended RNA impurities were reduced, which explains why dsRNA was further reduced when combining T7 RNAP G753A with m1ψ. Additionally, the yield of mRNA produced by T7 RNAP G753A catalyzing and using m1ψ as a raw material was similar to that obtained when catalyzed by the T7 RNAP WT (Table S1). Due to the reduced adverse impact on the yield of mRNA products without the use of harsh reaction conditions (high temperature) or added chemical reagents, T7 RNAP G753A binding with m1ψ has greater economic production significance as it significantly reduces dsRNA production levels. Except for removing dsRNA byproducts, the reliability of the replacement is also an important issue for the development of applications based on mRNA [56]. This analysis is beyond the scope of this study, but it will be studied in the near future.

### 2.4. Substitution Produced More Fragmented mRNA

**Figure 5:**
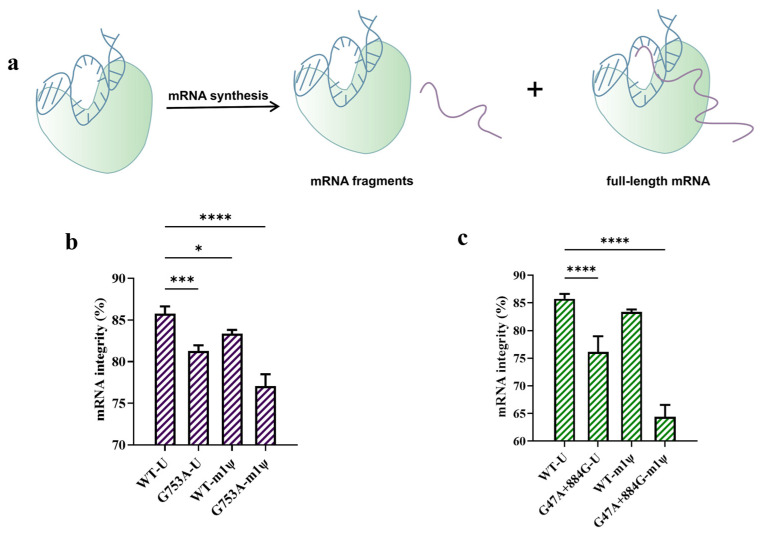
The performance testing of T7 RNAP and its variants (the error bars represent ±SD; * *p*  <  0.05, *** *p*  <  0.001, **** *p*  <  0.0001). (**a**) A schematic diagram showing the production of mRNA fragments transcribed in vitro. (**b**) The difference in mRNA integrity measured by CE (%). (**c**) G47A+884G generated more fragmented mRNA in in vitro transcription (%).

The largest proportion of impurities—namely fragmented mRNA products—can be produced during in vitro transcription [18] (Figure 5a). We measured mRNA integrity using capillary electrophoresis (CE); as shown in Figure 5b, replacing the T7 RNAP WT with T7 RNAP G753A caused more fragmented mRNA to be produced. The proportion of targeted mRNA was only 81.28 ± 0.69% among all mRNA products, while the integrity of mRNA catalyzed by the T7 RNAP WT was 85.75 ± 0.88%. When we replaced U with m1ψ as the substrate and T7 RNAP G753A as the catalyst, mRNA integrity was further decreased to 77.08 ± 1.41%, which demonstrates that the use of m1ψ exacerbated the formation of fragments, with more than 20% of mRNA products being fragmented (Figure 5b).

This fragmentation of mRNA mainly occurred in two ways: Mg^2+^-accelerated mRNA degradation [34,35,36,37,38] or mRNA products left the transcription complex early and then formed fragmented mRNA [33]. Based on the mechanisms of fragmented mRNA formation, we inferred that the G753 mutant reduced non-specific initiation and may have decreased affinity with the DNA template, thus generating fragmented mRNA. To further understand whether it was a coincidence or whether there was a correlation between dsRNA reduction and a decrease in mRNA integrity, we used another mutant, T7 RNAP G47A+884G, which was also reported to reduce dsRNA production [51]. As shown in Figure 5c, T7 RNAP G47A+884G sharply decreased mRNA integrity; when we used U as the substrate, the mRNA integrity was 76.15 ± 2.83%, and when we used m1ψ instead of U, the mRNA integrity sharply dropped to 64.40 ± 2.15%. Nearly one-third of the mRNA products were fragmented, which is much more than that transcribed by T7 RNAP G753A. Not only the T7 RNAP mutants, but also m1ψ slightly decreased mRNA integrity when using the T7 RNAP WT as the catalyst [33]. These results demonstrate that the most effective methods of reducing dsRNA production in in vitro transcription may have a negative effect on mRNA integrity, but the mechanism is not very clear.

**Figure 6:**
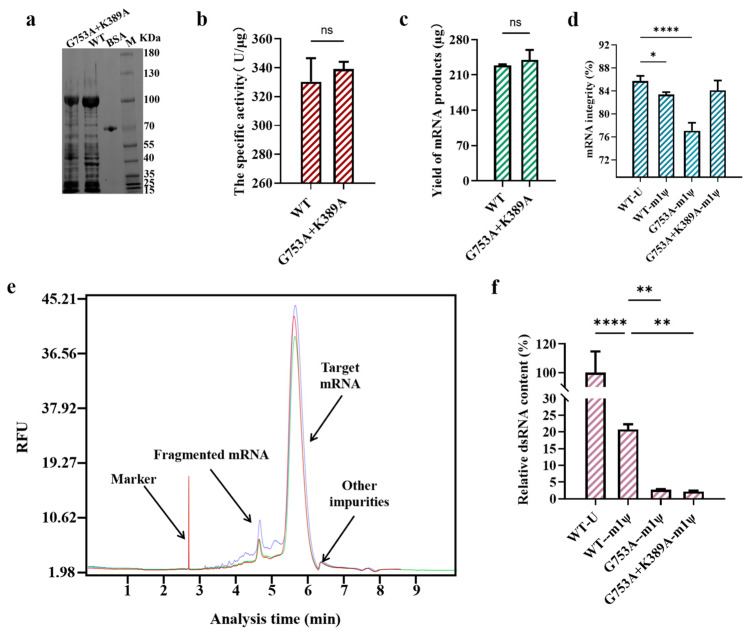
The performance testing of T7 RNAP G753A+K389A (the error bars represent ±SD; * *p*  <  0.05, ** *p*  <  0.01, **** *p*  <  0.0001). (**a**) An SDS-PAGE analysis of the overexpression of the protein. (**b**) The measurement of the specific activity of enzymes. (**c**) The yield of mRNA products. (**d**) The difference in mRNA integrity (%). (**e**) A CE analysis of mRNA products (green: mRNA products transcribed by the T7 RNAP WT; blue: mRNA products transcribed by T7 RNAP G753A; red: mRNA products synthesized via T7 RNAP G753A+K389A). (**f**) The relative dsRNA content (%).

He et al. found that using T7 RNAP K389A-catalyzed mRNA synthesized high-integrity mRNA products when using m1ψ as the substrate, which means the combination with the K389A mutant may have the potential to eliminate the negative impact on mRNA integrity when controlling dsRNA production in in vitro transcription [33]. After binding K389A with the G753 mutant, the target protein expression and the yield of mRNA products were not impacted, and the specific activity was improved slightly, although it was not significant (Figure 6a–c). Furthermore, the mutant group (G753A+K389A) kept the advantage of the single mutation (K389A) which significantly reduced fragmented mRNA production and improved mRNA integrity to 85.98 ± 0.93% (when U was used as the substrate) and 84.10 ± 1.75% (when m1ψ was used as the substrate) (Figure 6d,e and Table S1). After increasing the full-length RNA proportion by nearly 7%, the ratio of full-length mRNA among all mRNA products catalyzed by T7 RNAP G753A+K389A (m1ψ as the substrate) was similar to that obtained with the T7 RNAP WT (85.75 ± 0.88% with U as the substrate; 83.38 ± 0.44% with m1ψ as the substrate). In addition, there was no negative effect on reducing dsRNA impurities, and dsRNA production decreased by more than 97% in in vitro transcription catalyzed by T7 RNAP G753A+K389A (Figure 6f). This may be due to the increased stability of the transcription complex by including K389A, but it has no effect on non-specific recognition initiation. Overall, the combination of K389A with G753A is an effective method to eliminate the negative influence on mRNA integrity and keep the advantage of dsRNA decrease.

## 3. Materials and Methods

### 3.1. Reagents and Chemicals

Kits, including the high-yield RNA transcription kit, dsRNA quantitative kit, site-directed mutagenesis kit, and BCA Protein Assay Kit, and enzymes including murine RNase inhibitor (RI), Pyrophosphatase (PP), DNase I, and nucleic acids (A/G/C/UTP) were supplied by Vazyme Co., Ltd. (Nanjing, China). MgCl_2_, dithiothreitol (DTT), and other chemicals were obtained from Aladdin Industrial Inc. (Shanghai, China) or other commercial sources.

### 3.2. Overexpression and Purification of T7 RNAP

The recombinant vector pQE-30 (Figure S1) containing a nucleotide sequence expressing the target protein T7 RNAP (a DNA sequence expressing His-tag inserted at the 5′ terminal of the target sequence) was electro-transformed into *Escherichia coli* BL21 (DE3) (Novagen Inc., (Madison, WI, USA)) (Supplementary SEQ ID NO.1 and 2). The cells were cultured in a Luria–Bertani medium containing 100 mM of ampicillin at 37 °C until the OD 600 reached 1.2 or 0.6~0.8. The overexpression of T7 RNAP was induced by the final concentration of 0.5 mM of IPTG and then underwent continuous fermentation overnight before being collected. Then, the cells were resuspended and lysed by sonication. The content of crude enzymes was analyzed using 8% SDS-PAGE (Smart-Lifesciences, Changzhou, China).

Subsequently, the supernatants were pretreated by adding a final concentration of 0.3% (*v*/*v*) of polyethyleneimine (PEI) after removing the impurities via centrifugation. The target protein was precipitated using ammonium sulfate with a saturation of 60%. Subsequently, the precipitation was resuspended with a Ni column binding buffer (10×, 3 M NaCl, 2 mM DTT, 5% *v*/*v* glycerol, 200 mM imidazole, 200 mM Tris-HCl, pH 7.5) and loaded into a His trap HP (GE Healthcare). The column was eluted with the buffer containing a high concentration of imidazole (10×, 2 M NaCl, 2 mM DTT, 5% *v*/*v* glycerol, 4.5 M imidazole, 200 mM Tris-HCl, pH 7.5). Finally, the purified T7 RNAP was dialyzed against the dialysis buffer (10×, 500 mM Tris-HCl, 50% *v*/*v* glycerol, 1M NaCl, 10 mM DTT, 1 mM EDTA-2Na, pH 8.0). The target protein was quantified using the BCA method.

### 3.3. The Measurement of Specific Activity

The activities of the samples (T7 RNAP WT/mutants) were estimated, and the samples were diluted to be the same as the activity of the standard T7 RNAP (Vazyme Co., Ltd., Nanjing, China). The standard and samples were then gradient-diluted using the buffer (10×, 500 mM Tris-HCl, 1M NaCl, 1 mM EDTA-2Na, and 10% *v*/*v* glycerol, pH 8.0). The reaction of the activity measurement contained 0.5 mM of A/G/C/UTP, 2 U/μL of RI, 20% *v*/*v* samples, and 4 ng/μL of the linear DNA template (Supplementary SEQ NO.3), and the reaction mixture was incubated at 37 °C for half an hour. Subsequently, the reaction was mixed with nucleic acid dye BR reagent, and then, quantitation was performed using fluorescence detection with an excitation wavelength of 630 nm and an emission wavelength of 680 nm; the gain value was set as 126. According to the linear regression equation (slope = k), the activity was defined as follows:

Y (Standard) = k_1_X + b (1)

Y (Sample) = k_2_X + b (2)

Activity of sample = k_2_/k_1_ × Estimated value of activity (U/μL) (3)

(4) $$\mathrm{Specific}\;\mathrm{activity}\;\mathrm{of}\;\mathrm{sample}=\frac{\mathrm{Activity}\;\mathrm{of}\;\mathrm{protein}\;(U/\;\mu L)}{\mathrm{Concentration}\;\mathrm{of}\;\mathrm{protein}\;(\mu g/\mu L)}$$

### 3.4. Measurement of dsRNA Content

The dsRNA quantitative kit was used to measure the dsRNA content in the mRNA products transcribed in vitro. The procedure was as follows: The calibrator (300 ng/mL) were diluted to 0.75, 0.375, 0.187, 0.093, 0.047, and 0.023 ng/mL. A volume of 100 μL of the samples was gradient-diluted and added onto an Elisa plate. After sealing the plate with a sealing membrane, it was incubated at 37 °C for 60 min. The sealing membrane was then carefully removed, and the liquid was discarded. An amount of 300 μL 1 × scrubbing solution was added to each well and left to stand for 30 s. This step was repeated 4 times to remove residual liquid. An amount of 100 μL 1 × detection antibodies (Cat: DD3509-01-AE, Vazyme Co., Ltd. (Nanjing, China)) (diluted with the 100 × detection antibody) was added to each well, and the plate was incubated at 37 °C for an hour and then washed as before. Each well contained 100 μL of the enzyme-labeled antibody (Cat: DD3509-01-AG, Vazyme Co., Ltd. (Nanjing, China)) (diluted with the 100 × enzyme-labeled antibody). After incubation at 37 °C for 60 min, the plate was washed again. Subsequently, 100 μL of colorimetric solution was added to each well. The lightproof reaction was carried out for 15 min at 37 °C and terminated by adding 50 μL of termination solution. Thus, the dsRNA yield was measured using a microplate reader with an excitation wavelength of 450 nm.

### 3.5. An Analysis of Antisense RNA Production Transcribed Using DNA as the Template

The reaction of antisense RNA synthesis using DNA as the template contained 10 mM of A/G/C/UTP, 2 U/μL of RI, 50 ng/μL of the DNA template (target sequence, Supplementary SEQ NO.4), 0.005 U/μL of PP, 20 U/μL of the T7 RNAP WT and mutants, 22.5 mM of MgCl_2_, 2.5 mM of Tcep, 2 mM of Spermidine, and 40 mM of Tris-HCl at pH 8.0, and the reaction mixture was incubated at 37 °C for 60 min. Subsequently, the RNA products were digested by DNase I and then purified, and the products were diluted in RNase-free H_2_O (2.5 times the reaction mixture volume) and then analyzed using 6% SDS-PAGE (electrophoretic buffer: #1B548102 (Sangon Biotech Co., Ltd., Shanghai, China); running conditions: 160 V 30 min; dye of the PAGE gel: Untra GelRed).

### 3.6. An Analysis of Antisense RNA Production Transcribed Using RNA as the Template

Similarly, the reaction of antisense RNA synthesis using RNA (5′-CCAAAAUUUCAAGAUCAGGGCUUGAAAUUUUGUAAAAUUUCAAGCCCUGAUCUUGAA AUUUUCCA-3′, GenScript, Nanjing, China) as the template contained 7.5 mM of A/G/C/UTP, 2 U/μL of RI, 0.005 U/μL of PP, 50 ng/μL of the RNA template, 20 U/μL of the T7 RNAP WT and mutants, 30 mM of MgCl_2_, 2.5 mM of Tcep, 2 mM of Spermidine, and 40 mM of Tris-HCl at pH 8.0. The reaction mixture was incubated at 37 °C for 15 h. Subsequently, RNA products were obtained and then further purified. The purified products were diluted in RNase-free H_2_O (2.5 times the reaction mixture volume) and then analyzed using 12% SDS-PAGE (electrophoretic buffer: #1B548102 (Sangon Biotech Co., Ltd., Shanghai, China); running conditions: 160 V 30 min; dye of the PAGE gel: Untra GelRed).

### 3.7. 3′-Overextended mRNA Measured Using LC-MS

The compositions of the in vitro transcription reaction mixture for measuring the dsRNA generated by 3′ excessive elongation are as follows: 50 ng/μL of DNA template (Supplementary Sequence NO.5, GenScript, Nanjing, China); 10 mM each of A/G/C/UTP; 2 U/μL of RI; 0.005 U/μL of PP; 15 U/μL of T7 RNAP; 40 mM of Tris-HCl; 30 mM of magnesium chloride; 2 mM of spermidine; and 2.5mM of Tcep at pH 8.0. The reaction mixture was incubated for 60 min at 37 °C to allow transcription to occur. Then, the RNA products were treated with DNase I to digest any remaining DNA template, and the RNA products were purified using RNA clean magnetic beads to remove contaminants. The yield of the RNA products was quantified using a Nanodrop 2000C spectrophotometer. Finally, the purity of the RNA products was assessed using liquid chromatography–mass spectrometry (LC-MS) (Thermo scientific Vanquish Flex-Qrbitrap Exploris120, column: ChromCore C18, 1.8 μm (dimension: 4.6 × 50 mm); column temperature: 60 °C; gradient elution solvents: solvent A (0.065% HFIP and 0.0375% DIPEA in ddH₂O) and solvent B (0.065% HFIP and 0.0375% DIPEA in MeOH); flow rate: 0.3 mL/min; detection: a full scan was performed within the range of 600–3000 *m*/*z* and detection analyses were carried out in negative ion mode). mRNA integrity and the yield of mRNA products are shown in Table S1 and Figure S2.

## 4. Conclusions

This study explored the mechanisms of dsRNA impurity generation during in vitro transcription by T7 RNAP. By targeting the specificity loop, specifically the G753A mutation, we significantly reduced dsRNA production to 17.76 ± 1.37% of its initial content. This mutation likely impacted the stability of non-specific binding, thereby reducing antisense RNA production. Additionally, combining the G753A mutation with m1ψTP substrates further decreased relative dsRNA impurities to <3%, demonstrating a synergistic effect. Even though the replacement of G753A produced more fragmented mRNA, adding K389A effectively eliminated the adverse impact. Overall, these findings contribute to the development of more efficient and safer mRNA-based therapies, potentially improving their clinical outcomes.

## Data Availability

The data presented in this study are available in the article and Supplementary Materials.

## Supplementary Materials

The following supporting information can be downloaded at https://www.mdpi.com/article/10.3390/molecules29194713/s1: Figure S1: Map of recombinant plasmid pQE-30 of T7 WT; Figure S2: CE analysis of mRNA integrity transcribed by T7 RNAP G47A+884G; Table S1: Performance of T7 RNAP WT and mutants; SEQ NO.1: optimized cDNA sequence of T7 RNA WT; SEQ NO.2: amino acid sequence of T7 RNAP WT; SEQ NO.3: original DNA template sequence; SEQ NO.4: 512B sequence; SEQ NO.5: 30 nt.
