# Supplementary material for: Effective Synthesis of mRNA during In Vitro Transcription with Fewer Impurities Produced
Source: Molecules. 2024 Oct 5;29(19):4713. doi: 10.3390/molecules29194713 (PMC11477551; doi:10.3390/molecules29194713)
Supplement: Supplementary file 1 [file molecules-29-04713-s001.zip › molecules-3182319-supplementary.pdf]

Figure Captions

Figure S1. Map of recombinant plasmid pQE-30 of T7 WT.
Figure S2. CE analysis of mRNA integrity which transcribed by T7 RNAP G47A+884G.

Table Captions

Table S1. mRNA integrity and the yield of mRNA products (The experiments were replicated $\geq$ 3 times; error bars represent ±SD).

Sequence Captions

SEQ NO.1. Optimized cDNA sequence of T7 RNA WT.
SEQ NO.2. Amino acids sequence of T7 RNAP WT.
SEQ NO.3. Original DNA template sequence.
SEQ NO.4. 512B sequence (5'-3').
SEQ NO.5. 30 nt.

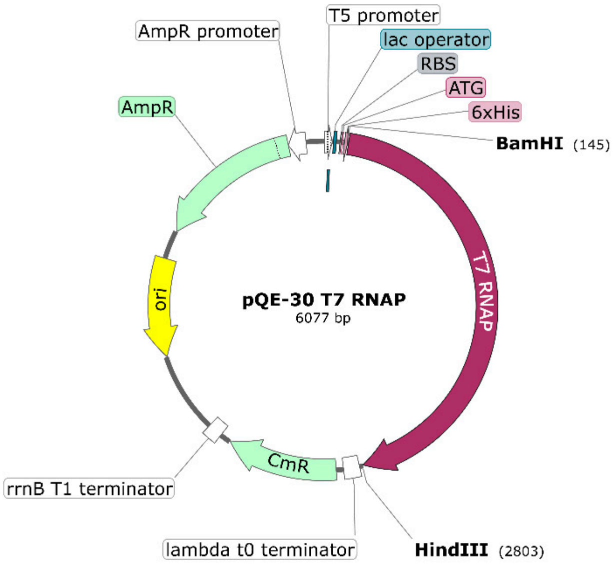

Figure S1. Map of recombinant plasmid pQE-30 of T7 WT.

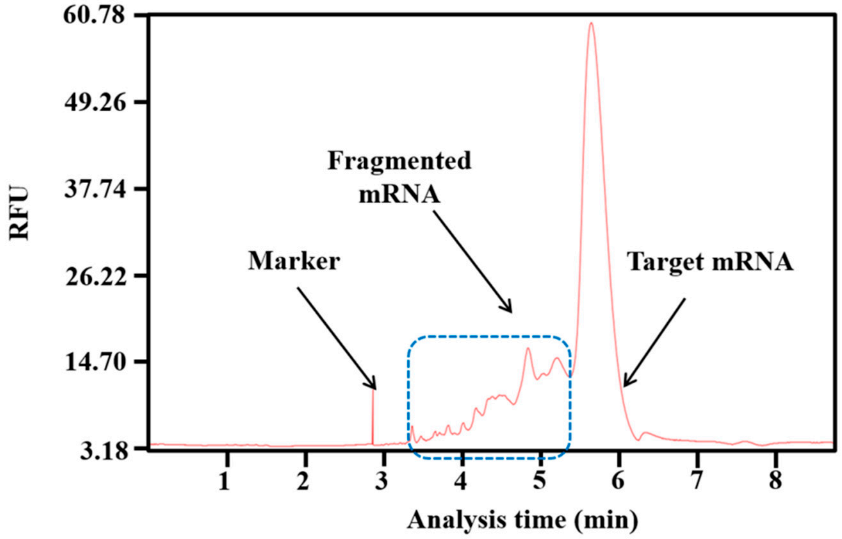

Figure S2. CE analysis of mRNA integrity which transcribed by T7 RNAP G47A+884G.

Table S1. mRNA integrity and the yield of mRNA products.
(The experiments were replicated ≥ 3 times; error bars represent ±SD).

Substitutions	mRNA Integrity (%)	Yield of mRNA products (μg)	Relative dsRNA content (%)
WT-U	85.75 ± 0.88	228.80±2.34	100.00±14.78
G753A-U	81.28±0.69	230.69±5.24	17.76±1.37
G753M-U	62.00±0.42	220.20±3.17	189.70±20.27
G753N-U	82.85±1.31	221.93±30.20	22.04±2.81
G753C-U	74.73±3.87	137.39±3.71	29.39±2.06
G753Q-U	80.98±0.25	228.73±6.48	21.67±2.02
G753W-U	39.90±0.85	190.88±7.65	63.91±7.66
T763A-U	84.15±0.07	226.75±3.87	677.30±65.36
G47A+884G-U	76.15±2.83	45.72±27.13	/
G753A+K389A-U	85.98±0.93	239.62±19.96	20.12±1.81
WT-m1Ψ	83.38±0.44	227.84±4.50	20.76±1.53
G753A- m1Ψ	77.08±1.41	223.63±11.47	2.74±0.18
G753A+K389A- m1Ψ	84.10±1.75	224.03±10.17	2.20±0.26

Sequences

SEQ NO.1. Optimized cDNA sequence of T7 RNA WT

ATGAATACAATTAACATAGCTAAAAATGATTTCAGCGATATTGAACTGGCTG
CGATCCCGTTTAATACCTTGGCGGACCATTATGGTGAGCGTCTGGCGCGTGA
GCAACTTGCTCTGGAGCACGAATCCTACGAGATGGGTGAAGCGCGTTTCCG
GAAGATGTTCGAGCGCCAGCTTAAGGCTGGCGAGGTGGCGGATAACGCTG
CTGCGAAGCCGTTGATTACGACCCTGCTGCCGAAAATGATTGCGCGTATTA
ACGATTGGTTCGAGGAAGTGAAAGCAAAGCGTGGTAAACGTCCAACCGCA
TTCCAGTTTCTGCAAGAGATCAAGCCGGAGGCGGTGGCATACATTACGATC
AAAACCACTCTCGCATGTCTCACCAGCGCGGATAATACTACGGTCCAAGCG
GTGGCCAGCGCGATTGGCCGCGCGATTGAGGACGAAGCGCGTTTTGGTCG
CATTCGTGACCTGGAAGCGAAACATTTTAAGAAGAACGTTGAAGAACAAT
TGAATAAACGCGTGGGCCACGTTTACAAGAAAGCGTTCATGCAAGTCGTTG
AAGCTGATATGCTGAGCAAAGGCCTGTTAGGTGGTGAAGCATGGAGCAGC
TGGCATAAGGAGGACTCAATTCACGTCGGCGTGCGGTGCATTGAAATGCTG
ATCGAATCGACCGGTATGGTTAGCCTGCATCGCCAAAACGCCGGCGTGGTG
GGTCAAGATAGCGAAACGATTGAACTGGCGCCAGAGTACGCGGAAGCTAT
CGCTACCAGAGCCGGCGCATTGGCGGGGATCAGCCCGATGTTCCAGCCGTG
TGTTGTACCACCGAAGCCGTGGACCGGTATCACCGGTGGAGGTTACTGGGC
GAATGGTAGACGTCCGTTGGCGTTGGTTCGTACCCATAGCAAAAAGGCTCT
GATGCGTTATGAAGACGTTTACATGCCGGAAGTGTACAAAGCCATCAACAT
CGCGCAGAACACTGCGTGGAAAATCAACAAAAAGGTGTTAGCTGTGGCGA
ACGTAATTACAAAATGGAAGCACTGCCCGGTTGAGGACATCCCGGCTATTG
AACGTGAAGAGCTGCCAATGAAACCAGAGGACATTGACATGAATCCGGAG
GCGCTGACCGCATGGAAACGTGCGGCAGCAGCGGTCTATCGTAAGGATAA
AGCCAGAAAGAGCCGTCGTATCTCCCTGGAATTCATGCTTGAGCAGGCGAA
CAAATTCGCTAACCACAAAGCCATCTGGTTTCCGTATAATATGGATTGGCGT
GGTCGTGTTTACGCGGTATCGATGTTCAACCCGCAGGGTAATGACATGACC
AAGGGTTTGTTGACCTTGGCCAAAGGTAAACCGATTGGCAAAGAAGGTTA
CTATTGGTTGAAGATCCACGGTGCGAACTGCGCAGGGGTGGATAAGGTACC
GTTCCCGGAACGTATTAAATTCATCGAGGAAAACCACGAAAACATTATGGC
GTGCGCAAAGAGCCCGCTGGAAAACACGTGGTGGGCAGAGCAGGACAGT
CCATTCTGCTTCTTGGCGTTCTGTTTCGAATATGCAGGTGTCCAGCACCACG
GCCTGAGCTATAACTGCAGCCTGCCGCTGGCCTTTGATGGTTCTTGTAGCG
GTATTCAACACTTTAGCGCCATGCTGCGCGATGAGGTTGGTGGCAGAGCAG
TGAATCTGCTGCCAAGCGAAACCGTGCAAGATATCTATGGCATTGTGGCTA
AGAAGGTTAATGAAATCCTGCAAGCAGACGCGATTAACGGCACGGACAAC
GAGGTTGTCACCGTGACCGATGAAAACACCGGTGAGATCTCGGAGAAAGT
GAAACTGGGCACCAAAGCCTTGGCTGGCCAATGGCTGGCATACGGCGTTA
CTCGCTCCGTGACCAAGCGTTCGGTGATGACCCTTGCTTACGGCAGCAAAG
AGTTTGGTTTCCGCCAACAGGTTCTGGAGGATACTATCCAACCGGCCATCG
ACTCTGGTAAAGGCCTGATGTTCACGCAGCCGAATCAGGCAGCGGGCTATA
TGGCGAAGCTGATTTGGGAATCGGTTAGCGTTACCGTTGTTGCAGCGGTGG

AGGCTATGAACTGGCTGAAATCTGCGGCGAAGCTGTTAGCTGCGGAGGTG
AAGGACAAGAAGACGGGCGAAATCCTGCGTAAGCGCTGCGCAGTGCATTG
GGTTACGCCGGATGGTTTTCCGGTATGGCAGGAGTACAAAAAGCCTATTCA
GACCCGTCTGAACCTGATGTTTCTGGGTCAATTTCGTCTGCAGCCGACGAT
CAACACCAATAAGGACTCCGAGATCGACGCGCACAAACAGGAGTCTGGCA
TCGCACCCAATTTCGTTCACTCACAGGACGGTTCCCATCTGCGTAAGACCG
TCGTGTGGGCACACGAGAAGTATGGCATTGAGTCGTTTGCATTGATCCACG
ATTCCTTTGGTACCATCCCGGCGGACGCTGCGAACCTGTTTAAAGCGGTTC
GCGAAACCATGGTTGACACCTATGAAAGTTGCGATGTTCTGGCAGACTTTT
ATGATCAGTTCGCCGACCAACTGCATGAGTCCCAGCTGGATAAAATGCCGG
CGTTACCGGCGAAGGGCAACTTAAATCTCCGCGACATCCTGGAATCTGATT
TCGCCTTCGCGTAA

SEQ NO.2. Amino acids sequence of T7 RNAP WT
MNTINIAKNDFSDIELAAIPFNTLADHYGERLAREQLALEHESYEMGEARFRK
MFERQLKAGEVADNAAAKPLITTLLPKMIARINDWFEEVKAKRGKRPTAFQF
LQEIKPEAVAYITIKTTLACLTSADNTTVQAVASAIGRAIEDEARFGRIRDLEAK
HFKKNVEEQLNKRVGHVYKKAFMQVVEADMLSKGLLGGEAWSSWHKEDSI
HVGVRCIEMLIESTGMVSLHRQNAGVVGQDSETIELAPEYAEAIATRAGALAG
ISPMFQPCVVPPKPWTGITGGGYWANGRRPLALVRTHSKKALMRYEDVYMPE
VYKAINIAQNTAWKINKKVLAVANVITKWKHCPVEDIPAIEREELPMKPEDID
MNPEALTAWKRAAAAVYRKDKARKSRRISLEFMLEQANKFANHKAIWFPYN
MDWRGRVYAVSMFNPQGNDMTKGLLTLAKGKPIGKEGYYWLKIHGANCAG
VDKVPFPERIKFIEENHENIMACAKSPLENTWWAEQDSPFCFLAFCFEYAGVQ
HHGLSYNCSLPLAFDGSCSGIQHFSAMLRDEVGGRAVNLLPSETVQDIYGIVA
KKVNEILQADAINGTDNEVVTVTDENTGEISEKVKLGTKALAGQWLAYGVT
RSVTKRSVMTLAYGSKEFGFRQQVLEDTIQPAIDSGKGLMFTQPNQAAGYMA
KLIWESVSVTVVAAVEAMNWLKSAAKLLAAEVKDKKTGEILRKRCAVHWVT
PDGFPVWQEYKKPIQTRLNLMFLGQFRLQPTINTNKDSEIDAHKQESGIAPNF
VHSQDGSHLRKTVVWAHEKYGIESFALIHDSFGTIPADAANLFKAVRETMVDT
YESCDVLADFYDQFADQLHESQLDKMPALPAKGNLNLRDILESDFAFA

SEQ NO.3. Original DNA template sequence
TAATACGACTCACTATAGGGAGAAATAACAAATCTCAACACAACATATACA
AAACAAACGAATCTCAAGCAATCAAGCATTCTACTTCTATTGCAGCAATTTA
AATCATTTCTTTTAAAGCAAAAGCAATTTTCTGAAAATTTTCACCATTTACG
AACGATAGCAGCCACCATGTTCGTGTTTCTGGTGCTGCTGCCTCTGGTGAG
CTCCCAGTGCGTGAATCTGACCACAAGGACCCAGCTGCCCCCTGCCTATAC
CAACTCCTTCACACGGGGCGTGTACTATCCCGACAAGGTGTTCCGGAGCAG
CGTGCTGCACTCCACACAGGATCTGTTTCTGCCTTTCTTTTCTAACGTGACC
TGGTTCCACGCCATCCACGTGAGCGGCACCAATGGCACAAAGAGGTTCGA
CAACCCAGTGCTGCCCTTCAATGATGGCGTGTACTTCGCCTCCACCGAGAA
GTCTAATATCATCCGCGGCTGGATCTTTGGCACCACACTGGACAGCAAGAC
ACAGTCCCTGCTGATCGTGAACAATGCCACCAACGTGGTCATCAAGGTGTG

CGAGTTCCAGTTTTGTAACGATCCATTCCTGGGCGTGTACTATCACAAGAAC
AATAAGTCTTGGATGGAGAGCGAGTTTCGCGTGTATTCCTCTGCCAACAAT
TGCACATTTGAGTACGTGTCCCAGCCCTTCCTGATGGACCTGGAGGGCAAG
CAGGGCAATTTCAAGAACCTGCGGGAGTTCGTGTTTAAGAACATCGATGGC
TACTTCAAAATCTACTCCAAGCACACCCCAATCAATCTGGTGAGAGACCTG
CCACAGGGCTTCTCTGCCCTGGAGCCACTGGTGGATCTGCCCATCGGCATC
AACATCACCCGGTTTCAGACACTGCTGGCCCTGCACAGAAGCTACCTGAC
ACCAGGCGACAGCTCCTCTGGATGGACCGCAGGAGCAGCAGCCTACTATG
TGGGCTATCTGCAGCCCAGGACCTTCCTGCTGAAGTACAACGAGAATGGCA
CCATCACAGACGCAGTGGATTGCGCACTGGACCCCCTGTCTGAGACCAAG
TGTACACTGAAGTCCTTTACCGTGGAGAAGGGCATCTATCAGACAAGCAAC
TTCAGGGTGCAGCCTACCGAGTCCATCGTGCGCTTTCCCAATATCACAAAC
CTGTGCCCTTTTGGCGAGGTGTTCAATGCAACCAGGTTCGCAAGCGTGTAC
GCATGGAATAGGAAGCGCATCTCCAACTGCGTGGCCGACTATTCTGTGCTG
TACAATAGCGCCTCCTTCTCTACCTTTAAGTGCTACGGCGTGAGCCCCACAA
AGCTGAATGACCTGTGCTTTACCAACGTGTACGCCGATTCCTTCGTGATCA
GGGGCGACGAGGTGCGCCAGATCGCACCAGGACAGACAGGCAAGATCGC
AGACTACAACTATAAGCTGCCTGACGATTTCACCGGCTGCGTGATCGCCTG
GAACAGCAACAATCTGGATAGCAAAGTGGGCGGCAACTACAATTATCTGTA
CCGGCTGTTTAGAAAGTCTAACCTGAAGCCATTCGAGAGGGACATCTCCAC
AGAAATCTACCAGGCCGGCTCTACCCCCTGCAATGGCGTGGAGGGCTTTAA
CTGTTATTTCCCTCTGCAGAGCTACGGCTTCCAGCCAACAAATGGCGTGGG
CTATCAGCCCTACCGCGTGGTGGTGCTGTCTTTTGAGCTGCTGCACGCCCC
TGCAACAGTGTGCGGACCAAAGAAGTCCACCAATCTGGTGAAGAACAAGT
GCGTGAACTTCAACTTCAACGGACTGACCGGCACAGGCGTGCTGACCGAG
TCCAACAAGAAGTTCCTGCCTTTTCAGCAGTTCGGCAGGGACATCGCAGAT
ACCACAGACGCCGTGCGCGACCCTCAGACCCTGGAGATCCTGGATATCACA
CCATGCTCCTTCGGCGGCGTGTCTGTGATCACACCAGGCACCAATACAAGC
AACCAGGTGGCCGTGCTGTATCAGGACGTGAACTGTACCGAGGTGCCCGT
GGCAATCCACGCAGATCAGCTGACCCCTACATGGCGGGTGTACTCTACCGG
CAGCAACGTGTTCCAGACAAGAGCCGGATGCCTGATCGGAGCAGAGCACG
TGAACAATAGCTATGAGTGCGACATCCCTATCGGCGCCGGCATCTGTGCCTC
CTACCAGACCCAGACAAACTCCCCAAGGAGAGCACGGTCTGTGGCAAGCC
AGTCCATCATCGCCTATACCATGAGCCTGGGCGCCGAGAACTCCGTGGCCT
ACTCCAACAATTCTATCGCCATCCCTACCAATTTCACAATCTCCGTGACCAC
AGAGATCCTGCCAGTGAGCATGACCAAGACATCCGTGGACTGCACAATGTA
TATCTGTGGCGATTCCACCGAGTGCTCTAATCTGCTGCTGCAGTACGGCTCT
TTTTGTACCCAGCTGAACAGAGCCCTGACAGGCATCGCCGTGGAGCAGGA
CAAGAATACACAGGAGGTGTTCGCCCAGGTGAAGCAAATCTACAAGACCC
CACCCATCAAGGACTTTGGCGGCTTCAACTTTAGCCAGATCCTGCCCGATC
CTAGCAAGCCATCCAAGCGGTCTTTTATCGAGGACCTGCTGTTCAATAAGG
TGACCCTGGCCGATGCCGGCTTCATCAAGCAGTATGGCGATTGCCTGGGCG
ACATCGCCGCCAGAGACCTGATCTGTGCCCAGAAGTTTAACGGCCTGACCG
TGCTGCCTCCACTGCTGACAGATGAGATGATCGCCCAGTACACATCTGCCC

TGCTGGCAGGCACCATCACAAGCGGATGGACCTTCGGCGCAGGAGCCGCC
CTGCAGATCCCCTTTGCCATGCAGATGGCCTATCGGTTCAATGGCATCGGCG
TGACCCAGAATGTGCTGTACGAGAACCAGAAGCTGATCGCCAATCAGTTTA
ACTCCGCCATCGGCAAGATCCAGGACTCTCTGAGCTCCACAGCAAGCGCC
CTGGGCAAGCTGCAGGATGTGGTGAATCAGAACGCCCAGGCCCTGAACAC
CCTGGTGAAGCAGCTGTCTAGCAATTTCGGCGCCATCTCCTCTGTGCTGAA
CGATATCCTGAGCCGGCTGGACAAGGTGGAGGCAGAGGTGCAGATCGACC
GGCTGATCACAGGCAGACTGCAGTCCCTGCAGACCTACGTGACACAGCAG
CTGATCAgGGCAGCAGAGATCAGGGCATCTGCCAACCTGGCAGCAACCAA
GATGAGCGAGTGCGTGCTGGGCCAGTCCAAGAGAGTGGACTTTTGTGGCA
AGGGCTATCACCTGATGAGCTTCCCACAGTCCGCCCCTCACGGAGTGGTGT
TTCTGCACGTGACCTACGTGCCAGCCCAGGAGAAGAACTTCACCACAGCA
CCAGCAATCTGCCACGATGGCAAGGCACACTTTCCTAGGGAGGGCGTGTTC
GTGAGCAATGGCACCCACTGGTTTGTGACACAGCGCAACTTCTACGAGCC
ACAGATCATCACCACAGACAATACATTCGTGTCCGGCAACTGTGACGTGGT
CATCGGCATCGTGAACAATACCGTGTATGATCCTCTGCAGCCAGAGCTGGA
CTCTTTTAAGGAGGAGCTGGATAAGTACTTCAAGAACCACACCAGCCCCGA
CGTGGATCTGGGCGACATCTCTGGCATCAATGCCAGCGTGGTGAACATCCA
GAAGGAGATCGACAGGCTGAATGAGGTGGCCAAGAATCTGAACGAGTCCC
TGATCGATCTGCAGGAGCTGGGCAAGTATGAGCAGTACATCAAGTGGCCCT
GGTATATCTGGCTGGGCTTCATCGCCGGCCTGATCGCCATCGTGATGGTGAC
CATCATGCTGTGCTGTATGACAAGCTGCTGTTCCTGCCTGAAGGGCTGCTG
TTCTTGTGGCAGCTGCTGTAAGTTTGATGAGGACGATAGCGAGCCTGTGCT
GAAGGGCGTGAAGCTGCACTACACCTGAGAATTCAACCAGCCTCAAGAAC
ACCCGAATGGAGTCTCTAAGCTACATAATACCAACTTACACTTTACAAAATG
TTGTCCCCCAAAATGTAGCCATTCGTATCTGCTCCTAATAAAAAGAAAGTTT
CTTCACATTCTAACCAGCCTCAAGAACACCCGAATGGAGTCTCTAAGCTAC
ATAATACCAACTTACACTTTACAAAATGTTGTCCCCCAAAATGTAGCCATTC
GTATCTGCTCCTAATAAAAAGAAAGTTTCTTCACATTCTAAGCTTAAAAAAA
AAAAAAAAAAAAAAAAAAAAAAAAAAAAAAAAAAAAAAAAAAAAAAAAAA
AAAAAAAAAAAAAAAAAAAAAAAAAAAAAAAAAAAAAAAAAAAAAAAAAA
AAAAAAAAAAAAAAAAAAA

SEQ NO.4. 512B sequence (5’-3’)
TAATACGACTCACTATAGGGAGAAGCTCTCTTACACCTGATTCATTTCCATT
GTTTTCTGCAGCAGCAATCCGGTTTCTGTCTTCAATTGTCAACAGTTCCTCC
TCCATGCACTTATCCAAGACGTCTCTAACTAGAAGCTTGTCCACCAGAGTG
GGCTGAAGGAGGTTCAGCAGTTGGAGATATTCATCATGAGCGTTCTCAAAC
GATGGAGAGGGCAAGTCCGTGAGCTCAGGGTTCATGTAGCGGGCGGCCAG
AGGGCTGCCGGTTCTCCGGAGGGCCTCCACGAATTCCCGAGTCCAACCAA
GGTGCCAGACTCCCTTCTCCAAGGTGCTCAGCAGCAGTTCAACTGCCTGC
ATGTTCCCGGAGGTGGCGACTGTCCTCTGAATCTGCTCCTTCACCTCTGCA
GGCAGAAAGGTCAGGTAGTCCAGCACAGGCTCCACCTGGATGTACATTTTC
ACCCTGGCCCTGAAGCACGAGATGAGATAGCGGAAATTCTCGTCTGTGGA

ATACCCATT CGACATTCTCCC

SEQ NO.5. 30nt
CAGTAATACGACTCACTATAGATGATGATGATGATGATGATGATGATGAT
